# COVID-19 epidemic peaks distribution in the United-States of America, from epidemiological modeling to public health policies

**DOI:** 10.1038/s41598-023-30014-2

**Published:** 2023-03-27

**Authors:** Alexandre Vallée, Davide Faranda, Maxence Arutkin

**Affiliations:** 1 Department of Epidemiology-Data-Biostatistics, Delegation of Clinical Research and Innovation (DRCI), Foch Hospital, 92150 Suresnes, France; 2 Laboratoire des Sciences du Climat et de l’Environnement, CEA Saclay l’Orme des Merisiers, UMR 8212 CEA-CNRS-UVSQ, Université Paris-Saclay, IPSL, 91191 Gif-sur-Yvette, France; 3 London Mathematical Laboratory, 8 Margravine Gardens, London, W6 8RH UK; 4 Laboratoire de Météorologie Dynamique/IPSL, École Normale Supérieure, PSL Research University, Sorbonne Université, École Polytechnique, IP Paris, CNRS, 75005 Paris, France; 5 School of Chemistry, The Center for Physics and Chemistry of Living Systems, Tel Aviv University, 6997801 Tel Aviv, Israel

**Keywords:** Epidemiology, Infectious diseases

## Abstract

COVID-19 prediction models are characterized by uncertainties due to fluctuating parameters, such as changes in infection or recovery rates. While deterministic models often predict epidemic peaks too early, incorporating these fluctuations into the SIR model can provide a more accurate representation of peak timing. Predicting R0, the basic reproduction number, remains a major challenge with significant implications for government policy and strategy. In this study, we propose a tool for policy makers to show the effects of possible fluctuations in policy strategies on different R0 levels. Results show that epidemic peaks in the United States occur at varying dates, up to 50, 87, and 82 days from the beginning of the second, third, and fourth waves. Our findings suggest that inaccurate predictions and public health policies may result from underestimating fluctuations in infection or recovery rates. Therefore, incorporating fluctuations into SIR models should be considered when predicting epidemic peak times to inform appropriate public health responses.

## Introduction

Understanding the predictions at the different stages of the evolution of key epidemic indicators remains a major goal for policy makers and health professionals. The dynamics of human-to-human transmission risk is associated with many factors, including response measures and other temporal factors that could affect the trajectory of the epidemic^1,2^. It is essential to consider fluctuations of epidemiological parameters (e.g., variations in infection or recovery rates) in the modeling of the epidemic spread to better predict the epidemic peak date. It remains extremely challenging to provide an accurate epidemic scenario of an epidemic both because of the partial knowledge of the health status of the population and the variability of virus characteristics^3^. Previous studies investigated that COVID-19 prediction models are characterized by uncertainties resulting in fluctuating factors of the epidemic^3–5^. Daily fluctuations of recovery rates present a main role in peak epidemic timing based on Susceptible-Infected-Recovered (SIR) model dynamics. Using such a model with fluctuating control parameters, a previous work has shown that the infection counts follow a stochastic process with a log-normal distribution at an early stage, and that the epidemic peak is a random variable when considering that these parameters fluctuate. A form of the stochastic solution of the infection counts at an early stage of the epidemics was derived. With this framework, it has been shown that the deterministic models anticipate the epidemic peaks with respect to the stochastic model. In the latter, fluctuations of infection and recovery rates induce a realistic delay on the most probable and average date of the epidemic peak. Based on the data of the Italian regions, we previously explained in this country that the dispersion of the epidemic peak date can be modeled by including fluctuations in the control parameters in a stochastic SIR model^3^. This study aims to examine how daily fluctuations in infection and recovery rates affect the dynamics of a Susceptible-Infected-Recovered (SIR) model and the timing of the epidemic peak in the United States. The study will use data from three COVID-19 waves in the United States to analyze the impact of these fluctuations on the most probable and average peak time of the epidemic using a theoretical deterministic model. The basic reproduction number (R0) will also be considered in the analysis. The results of this study will provide insight into the role of infection and recovery rate fluctuations on the spread and peak of an epidemic, particularly we will show that the deterministic SIR model anticipates the peak with respect to the most probable and average peak time of the stochastic model.

## Methods

### Theoretical modeling of the most probable epidemic peak time modeling

#### Deterministic SIR model

We build our analysis by showing that daily fluctuations in the infection and recovery rate are essential to improve prediction of the epidemic peak date and suggesting that it should be introduced in epidemiological models^3^. The epidemic peak date distribution allows for a new estimation of epidemic evolution using a Susceptible Infected Recovered (S.I.R) model with daily fluctuations on infection rates^6^.

The compartmental model divides the population into three groups, namely Susceptible (S), Infected (I), and Recovered (R) individuals, according to the following discrete-time evolution equations:

1 $$\frac{dS}{dt}=-\uplambda \frac{{I}_{t}{S}_{t}}{N}$$

2 $$\frac{dI}{dt}=-\uplambda \frac{{I}_{t}{S}_{t}}{N}-\upbeta\mathrm{It}$$

3 $$\frac{dR}{dt}=\upbeta\mathrm{It}$$

In the SIR model above, the parameters are the recovery rate (β), and the infection rate (λ), N is the total population.

At the beginning of the epidemic the number of susceptible people is considered constant (S ∼ N = constant) and large with respect to the number of infected people, without parameter fluctuation, we recover the exponential growth at early stage:

4 $$ {\text{I}}({\text{t}}) \sim {\text{I}}0 \exp ((\uplambda - \upbeta ) {\text{t}}) $$

this solution shows that if λ ≤ β or R_0_ = $\frac{\uplambda }{\upbeta }$≤ 1 there is no epidemic outbreak, this is called the epidemic threshold and exhibit the importance of the R_0_ to understand and control an epidemic dynamic^7–10^.

To consider time-dependent control factors, a stochastic approach is performed by which the control parameters $k\in \{\beta ;\lambda \}$ are described through a stochastic process:

5 $${k}_{t}={k}_{0}(1+{\sigma }_{k}{\epsilon }_{k,t})$$

where $\epsilon$ is a reduced centered Gaussian random variable. ${k}_{0}\in \{{\beta }_{0} ;{\lambda }_{0}\}$ is set to the mean value of the parameter. We show that the infection counts follow a log-normal distribution and then, we can investigate the quantile of this solution^11^. The log-normal distribution of the number of infected people implies sub-exponential divergence of the quantile of the solution from the average exponential growth behavior. Therefore, effectively managing an epidemic over a specific time frame and with a desired level of certainty should focus on managing a specific quantile of the solution Empirically we may consider the dynamic for the worst α (= 95% for example) scenarios of the solution. The corresponding α−quantile q_α_ for the Brownian motion is:

6 $${q}_{\alpha }\left(t\right)={\sqrt{2t}\text{ erf}}^{-1}\left(2\alpha -1\right)$$

Thus, the quantile of the number of infected people reads:

7 $$ I_{\alpha } \left( t \right) = I_{0} \exp \left( {\left( {m - \frac{{\tilde{\sigma }^{2} }}{{2\left( {1 + m} \right)^{2} }}} \right)t + \frac{{\tilde{\sigma }}}{{\left( {1 + m} \right)}}q_{\alpha } \left( t \right)} \right) $$

The log-normal distributions are positively skewed with long right tails due to low mean values and high variances in the random variables. This feature creates a balance between highly diffuse behavior at short time and drift domination at large time. A non-trivial time analogous to the time horizon appears canceling the exponent:

8 $$ T^{*} \left( \alpha \right) = \frac{{4\frac{{\tilde{\sigma }^{2} }}{{\left( {1 + m} \right)^{2} }}erf^{ - 1} \left( {2\alpha - 1} \right)^{2} }}{{2m - \frac{{\tilde{\sigma }^{2} }}{{\left( {1 + m} \right)^{2} }}}} $$

with m = λ_0_ – β_0_ and $${\tilde{\sigma }}\sqrt {{{\upbeta }}_{0} ^{2} {{\upsigma }}_{{{\upbeta }}} ^{2} + {{\uplambda }}^{2} _{0} {{\upsigma }}_{{{\uplambda }}} ^{2} }$$.

Thus, numerical experiments are performed by discretizing a SIR model defined previously with an Euler scheme and a time step $\Delta t=1$ day following the guideline defined by Faranda and Alberti^4^.

### Most probable date of the epidemic peak

The incorporation of fluctuating parameters in the SIR model introduces a level of randomness to the solution, making the predicted epidemic peak date itself a random variable We will use tool borrowed from first passage time theory to compute the probability distribution of the epidemic peak date, the first passage modeling has shown to be ubiquitous in nature: diffusion-limited growth^12^, neuron firing^13^, survival probability of a noble’s man name (male descendent)^14^, or the triggering of stock options^13^. At the epidemic peak, the number of infected people has reached its maximum, as an approximation, we will use the deterministic peak level from the SIR model and compute the random time at which the number of infected people reaches that level.

Assuming that the number of infected people at t = 0 is 1, for the deterministic SIR, an approximation of the number of infected people at the epidemic peak is^10,15^:

9 $${I}_{peak}\backsim N\left(1-\frac{1+\mathrm{log}\left({R}_{0}\right)}{{R}_{0}}\right)$$

using this approximation, the deterministic peak from the SIR model occurs at:

10 $${t}_{d}=\frac{\theta }{m}$$

with θ = log (I_peak_) and m = β_0_(R_0_ − 1). Now let’s assume that we introduce the fluctuation on the control parameters of the epidemic, the time at which the number of infected people will reach the level defined by Eq. (9) is a random variable defined as:

11 $$ {\text{t}}_{{{\text{peak}}}} = \mathop { \inf }\limits_{t} \left( {at + bW_{t} = \theta } \right) $$

with $a=(m-\frac{{\sigma }^{2}}{2{\left(1+m\right)}^{2}})$, $b=\frac{\sigma }{(1+m)}$, and ${W}_{t}$ the Brownian motion. The probability distribution of this random variable is equivalent to the first passage time distribution of a lognormal process to a given threshold^16^:

12 $$f\left({t}_{peak}\right)=\frac{\theta }{b\sqrt{2\pi {t}^{3}}}\mathrm{exp}\left(-\frac{{\left(\theta -at\right)}^{2}}{2{b}^{2}t}\right)$$

this time strongly depends on R_0_ and is delayed with respect to the deterministic peak time due to the fluctuations. Another interesting time to study is the most probable date of the epidemic peak:

13 $${t}_{mp}=\frac{{3b}^{2}}{{2a}^{2}}\left[\sqrt{1+\frac{{P}_{e}^{2}}{9}}-1\right]$$

where ${P}_{e}=\frac{2a\theta }{{b}^{2}}$ is the Péclet number of the model. When fluctuation play a major role—R_0_ ≃ 1—the most probable date for the epidemic peak scale like ${t}_{mp}=\frac{{t}_{mean}{P}_{e}}{6}$ with P_e_ → 0, and could be much smaller than average peak date of the epidemic. It is true particularly for R_0_ close to 1.

Thus, the epidemic peak time of the stochastic solution exhibits an inverse Gaussian probability distribution, that we will use to fit the spread of the epidemic peak times observed across the different regions/states^3^.

We recall that the probability distribution of the epidemic peak time is described by the following distribution:

14 $$f\left({t}_{peak}\right)=\frac{\theta }{b\sqrt{2\pi {t}^{3}}}\mathrm{exp}\left(-\frac{{\left(\theta -at\right)}^{2}}{2{b}^{2}t}\right)$$

with $a=(m-\frac{{\sigma }^{2}}{2{\left(1+m\right)}^{2}})$, $b=\frac{\sigma }{(1+m)}$, $m={\beta }_{0}({R}_{0}-1)$, ${\beta }_{0}$ is the recovery rate and $\sigma$ the amplitude of daily fluctuations of the control parameters (i.e., variations of infection rates or recovery rates). As shown above, this probability distribution one can easily get the most probable peak time, mean peak time and the confidence intervals of the epidemic peak time^3^.

### Data analysis

Epidemic peak time, when the outbreak reaches its highest point, is crucial for controlling the spread of the disease. From a modeling perspective, the distribution of epidemic peak time can be derived analytically using the following approximations: we assume that the epidemic peak time is determine by a drifted lognormal distribution to the deterministic peak level, as see above, for full probability distribution; the average epidemic peak time t_mean_ and the most probable epidemic peak time t_mp_ (for three waves in the United States of America) are derived analytically. The epidemic peak is delayed due to control parameters fluctuations with the SIR model (with conditions specified as above and 1.1 < R0 < 2) and from the analytical predictions. To compare our theoretical model to real data, we consider the U.S. states' infection counts. The data that support the findings of this study are openly available in https://github.com/CSSEGISandData/COVID-19. For each state, each epidemic wave started after the lowest number of infections between the previous wave to the next wave. The empirical distribution function of the epidemic peak time for each wave is fitted using maximum likelihood estimates of the theoretical epidemic peak time distribution defined above. Population of each US states came from: https://www.census.gov/data/tables/time-series/demo/popest/2020s-state-total.html#par_textimage. Analytic models were performed using MATLAB software.

## Results

**Figure 1:**
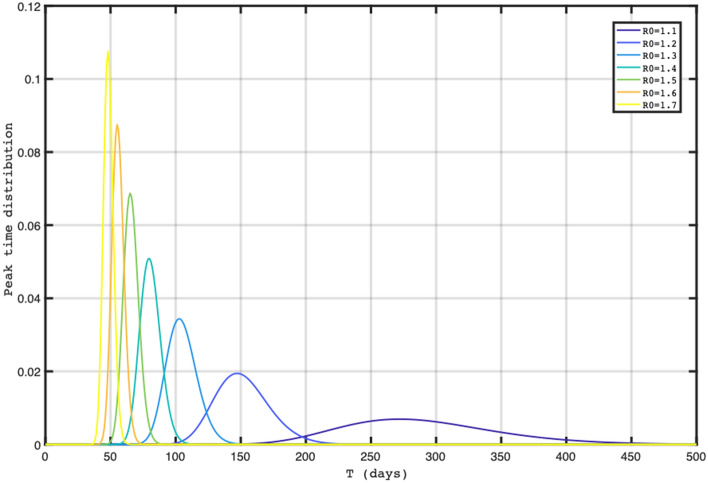
It illustrates the distribution of epidemic peaks at various intervals from the start of an outbreak, based on a theoretical model that considers R_0_ values ranging from 1.1 to 1.7. The figure displays how the peak of the epidemic may vary depending on the number of days since the beginning of the outbreak and the corresponding R_0_ value.

**Figure 2:**
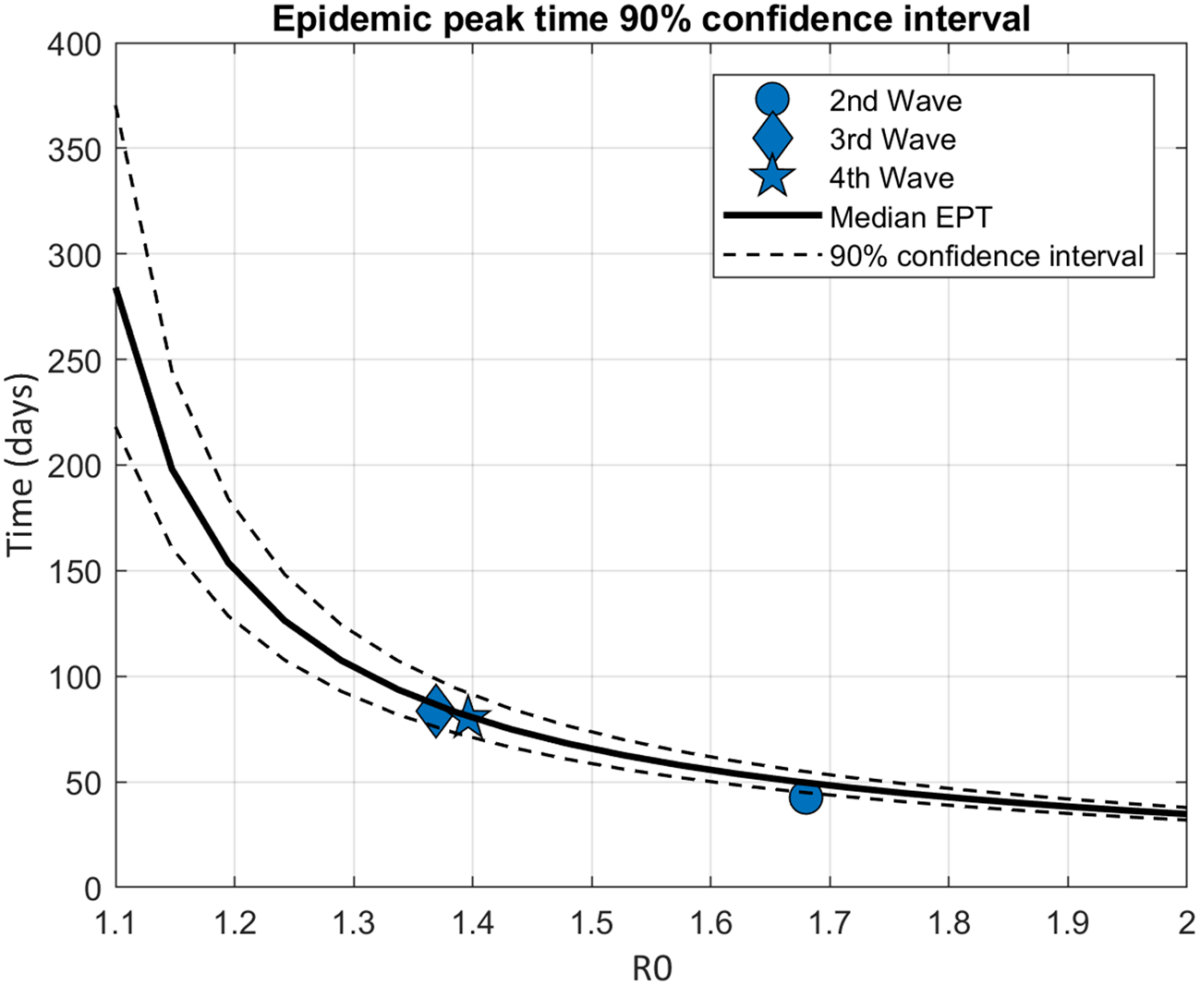
Median epidemic peak time for each wave and 90% confidence interval according to R_0_ level assuming control parameters daily fluctuations of 20%. EPT: epidemic peak time.

**Figure 3:**
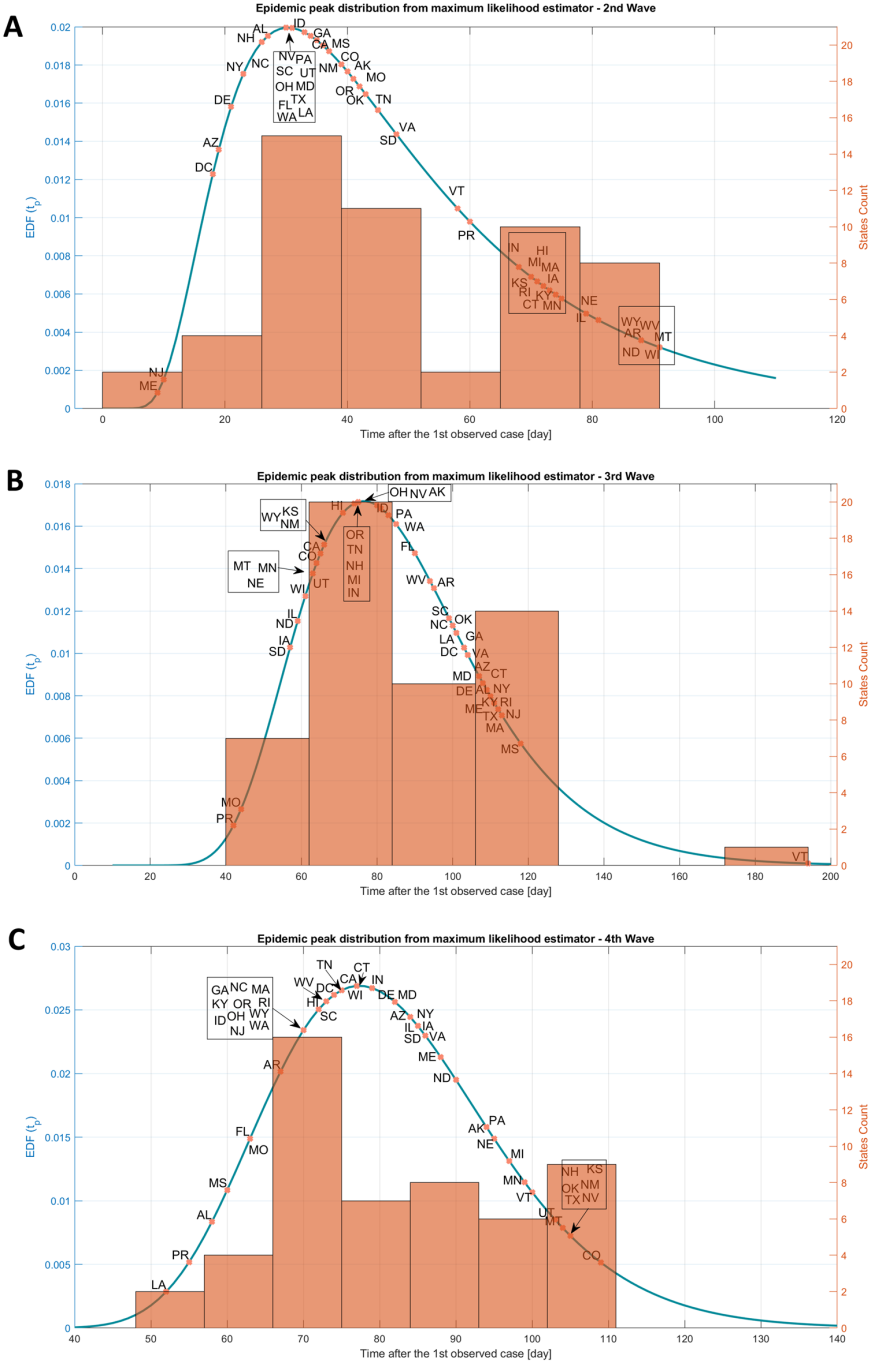
Epidemic peak distribution for the three last waves of the U.S. states, represented by alpha codes. Epidemic peak time is computed starting the time after the minimum of infection counts between two waves. The empirical distribution function of the epidemic peak is displayed in continuous lines, an histogram of the U.S. state counts date is shown in orange. (**A**) Epidemic peak distribution from maximum likelihood estimator for the second wave. (**B**) Epidemic peak distribution from maximum likelihood estimator for the third wave. (**C**) Epidemic peak distribution from maximum likelihood estimator for the fourth wave.

Figure 1 showed the different epidemic peak distributions according to the days from the beginning of a wave with different R0 comprising between 1.1 and 1.7 based on the theoretical model. Figure 2 showed the median epidemic peak time for each wave and 90% confidence interval according to R0 level based on analytical predictions and assuming control parameters daily fluctuations (as variations in infection rate or recovery rates) of 20%, with R0 ~ 1.7 for the second wave and R0 ~ 1.4 the third and fourth waves. The closer the R0 fluctuations are to 1.0, the greater the epidemic peak distribution will be large, with a larger confidence interval, with 275 days for R0 equal to 1.1 and with less than 50 days for R0 < 1.7. We observed that the peak across US states, for the second wave, was distributed around 50 days (most probable peak date) (SD 24 days) (Fig. 3A), for the third wave around 87 days (SD 26 days) (Fig. 3B), and for the fourth wave around 82 days (SD 15 days) (Fig. 3C).

## Discussion

For policymakers and health professionals, forecasting key pandemic indicators in the short term, such as the reproduction number (R0) and the number of new cases, is a crucial goal. Accurate predictions can inform the implementation of effective response strategies and aid in the more efficient allocation of resources The trajectory of the COVID-19 pandemic depends on a number of factors, including the attributes of the virus (such as its transmissibility), the characteristics of the location (such as population density and transportation patterns), individual behaviors in response to the pandemic, and government actions^1,17–19^. Understanding how these factors influence the spread of the disease is essential for policymakers and health professionals as they work to develop effective strategies for managing the pandemic. By analyzing the interplay between these factors and the trajectory of the pandemic, policymakers and health professionals can better understand the drivers of the disease's spread and develop more effective response strategies.

These factors are correlated with a more linear growth of pandemics^20^ but were still investigated in dynamic models of the COVID-19 transmission. Our study showed that epidemic peaks across the US. states during the second, third and fourth waves were distributed around 50, 87 and 82 days from the beginning to the peak of the epidemic wake (Fig. 3). Thus, fluctuations in models should be considered in the epidemic modeling to predict the epidemic peak and plan appropriate public policies.

### Epidemiological implications for public health policies

The epidemic peaks distributions are genuine features of the COVID-19 epidemic, and they originate from the combination of initial conditions (the health status of the population at the beginning of an epidemic wave) and the inherent fluctuations of the parameters that, in the SIR model, can be represented by stochastic fluctuations. Our previous study reported that epidemic peaks across the Italian region during the first wave were distributed around 55 days (most probable peak date), and around 130 days for the second wave^3^. Other models have shown a 6-week (~ 42 days) errors for cumulative death below 10%^19^, a median absolute percentage error at 10 weeks (~ 70 days) of forecasting COVID-19 resurgence for the Institute for Health Metrics and Evaluation (IHME) SEIR model^21^.

Many hypotheses have been mentioned to explain the divergence between the predicted and observed epidemic peaks, many inaccuracies and incompleteness of available information^22^, difficulties in confirming large numbers of cases by specific tests, presence of asymptomatic cases and possible delays in diagnosis, lack of testing, individual behavioral responses^23^, seasonality, meteorological factor^17^, variant spread^24^, worse the situation for modeling the different scenarios.

Uncertainties in predicting the peak of a pandemic can affect the efficacy of health policies and strategies. These uncertainties may be due to errors in long-term forecasts and to variations in the mechanisms of viral transmission. The expected exponential growth of transmission may not always be observed, which can be attributed to government interventions as well as individuals' reactions to the epidemic, such as self-isolation and practicing social distancing. These behavioral responses are indeed associated with a sub-exponential growth of epidemics^20,23^. However, these observations remain difficult to implement in the dynamic modeling of SARS-CoV-2 transmission. Restrictions in activities, such as non-pharmaceutical interventions and non-physical distancing factors, may probably help to delay the epidemic peak by playing a part in mitigating potential spikes in cases, especially when physical distancing measures are relaxed^25^. However, this work included limitations with large uncertainties for the estimate R_0_.

The validity of such claims depends on the evidence to support the hypotheses regarding the impact of a policy on transmission^18^. Different dynamics can interact with these models and impact the predicted gross scale of the epidemic. Indeed, the public health policies, including precautionary measures and quarantine, modulate the possible trajectories of outbreak^26^. Uncertainty in peak and date sizes can be due to numerous factors, including stochasticity of early dynamics, heterogeneity of contact profiles, spatial variation, and dynamics of epidemiological parameters^8^. While strong control policies have been associated with inmate growth in cases where house-stay restrictions were unlikely to be the one-size-fits-all agreement, a gradual approach to restrictive measures could be of concern^27^.

The prediction of R_0_ remains a major epidemiological challenge with practical consequences due to it supports governments policies to develop rapid strategies to counteract the growth of the outbreak. In this study, we propose a policy-maker tool which show the consequences of possible fluctuations in policy strategies on different R_0_ levels (Fig. 2)^28^.

### Limitations

Our data were provided from a public data source and thus, were limited to the accuracy of their report.

## Conclusion

Our study suggests that the distribution of epidemic peaks across different regions of the United States during each wave is not solely determined by the mean infection and recovery rates, but also by the fluctuations in these rates. This is an important finding as it highlights the need to consider fluctuations in predictive models and public health policies in order to have a more accurate prediction of the epidemic peak time.

Inaccurate predictions of both epidemic scenarios and public health policies could be the consequence of an underestimation of these fluctuations. This means that without considering the fluctuations in the infection and recovery rates, the predictions of the epidemic peak time could be incorrect. This could lead to inadequate and ineffective public health policies, which in turn can lead to a failure in controlling the spread of the disease.

To address this issue, our study proposed a policy-maker tool that incorporates fluctuations in R0 into predictive SIR models. This tool could be a valuable resource for policymakers in developing rapid strategies and implementing appropriate public health policies in response to outbreaks. By incorporating fluctuations in R0, the tool would provide policymakers with a better understanding of the potential impacts of different policy strategies on the spread of a disease. This could allow them to make more informed decisions and effectively allocate resources to prevent or mitigate outbreaks.

Overall, we emphasize the importance of considering the fluctuations in infection and recovery rates in order to have a more accurate prediction of the epidemic peak time, and thus to have a better epidemic control. This is crucial for policymakers and health professionals to develop effective response strategies and allocate resources in a more targeted way.

## Data Availability

The data that support the findings of this study are openly available in https://github.com/CSSEGISandData/COVID-19.
